# Sorption Behavior and Mechanisms of Organic Contaminants to Nano and Microplastics

**DOI:** 10.3390/molecules25081827

**Published:** 2020-04-16

**Authors:** Fang Wang, Min Zhang, Wei Sha, Yidong Wang, Huizhi Hao, Yuanyuan Dou, Yao Li

**Affiliations:** 1 Tianjin Key Laboratory of Water Resources and Environment, Tianjin Normal University, Tianjin 300387, China; 1820080037@stu.tjnu.edu.cn (M.Z.); 1920080035@stu.tjnu.edu.cn (W.S.); wangyidong@tjnu.edu.cn (Y.W.); 2 College of Environmental Science and Engineering/Ministry of Education Key Laboratory of Pollution Processes and Environmental Criteria/Tianjin Key Laboratory of Environmental Remediation and Pollution Control, Nankai University, Tong Yan Road 38, Tianjin 300350, China; 2120180686@mail.nankai.edu.cn (H.H.); 18342281420@163.com (Y.D.)

**Keywords:** nanoplastics, microplastics, organic contaminants, sorption mechanism, influencing factors

## Abstract

Nano and microplastics (NPs/MPs) have received widespread attention in recent years. Because of their large specific surface area and hydrophobicity, NPs/MPs can adsorb various organic contaminants. This article gives a brief review of the sorption behavior of organic contaminants to NPs/MPs, summarizes the possible sorption mechanisms, and analyzes the influencing factors in the environment on the sorption behavior and mechanisms of NPs/MPs. The main mechanisms of sorption of organic contaminants to NPs/MPs are partitioning, surface sorption (hydrogen bonding, π–π interaction, electrostatic interaction, and van der Waals force), and pore filling. The sorption behavior of organic contaminants to NPs/MPs is not only affected by the properties of the NPs/MPs and the organic contaminants, but also by the solution chemistry, such as the pH, ionic strength, and dissolved organic matter.

## 1. Introduction

Plastics are used worldwide because they are cheap, lightweight, durable, and corrosion-resistant [1,2,3]. The global plastics output was 311 million tons in 2014 [4], and the global demand for plastics has increased by about 245 million tons each year [5]. The rapid growth in production and applications of plastics has also raised many scientific concerns [6,7].

Plastics break down into small pieces after they go into the environment, and can be defined by their diameters as nanoplastics (NPs, lower than 0.1 μm) [8] and microplastics (MPs, 1–5000 μm) [9]. In recent years, they have been detected in aquatic environments [10], sediments [11], territorial systems [12], foods [13], and even in the animal and human tissues [14,15]. Many studies have reported that NPs/MPs show strong sorption affinities to various organic contaminants due to their large specific surface area and hydrophobicity [16]. For example, Wang et al. [17] reported that polystyrene (PS) NPs/MPs showed strong sorption capacity to both phenanthrene and nitrobenzene, and the distribution coefficient of phenanthrene was higher than that of nitrobenzene for MPs. Velzeboer et al. [18] compared the sorption behaviors of polychlorinated biphenyls (PCBs) to PS NPs and polyethylene (PE) MPs, and found that the sorption affinity to PS NPs was 1−2 orders of magnitude higher than to PE MPs, which is likely because of the stronger aromaticity of PS NPs and its higher surface area−volume ratio compared to PE MPs. Thus, the properties of both NPs/MPs and organic contaminants can affect the sorption behavior.

The sorption behavior of organic contaminants to NPs/MPs can be affected by the solution chemistry, such as pH, ionic strength, dissolved organic matter (DOM), etc. For example, Guo et al. [19] found that as the pH increased from 3.0 to 7.0, the amount of tylosin (TYL) adsorbed on PS and polyvinylchloride (PVC) decreased. The positively charged TYL was adsorbed on the PS and PVC due to electrostatic attraction when the pH was below 7.1. Li et al. [20] found that the ionic strength could affect the electrostatic force strength, because the cations could compete with the organic contaminants for the sorption sites, so the sorption affinity may decrease under high ionic strength. Wu et al. [21] reported that DOM could decrease the sorption affinities of the organic contaminants to PE because of their high affinity to DOM.

This article aims to give a brief review of the sorption behavior of organic contaminants to NPs/MPs, summarize the possible sorption mechanisms, and analyze the influencing factors in the environment on the sorption behavior and mechanisms of NPs/MPs. An outlook on future research is also given.

## 2. Sorption Mechanisms of Organic Contaminants to NPs/MPs

**Figure 1:**
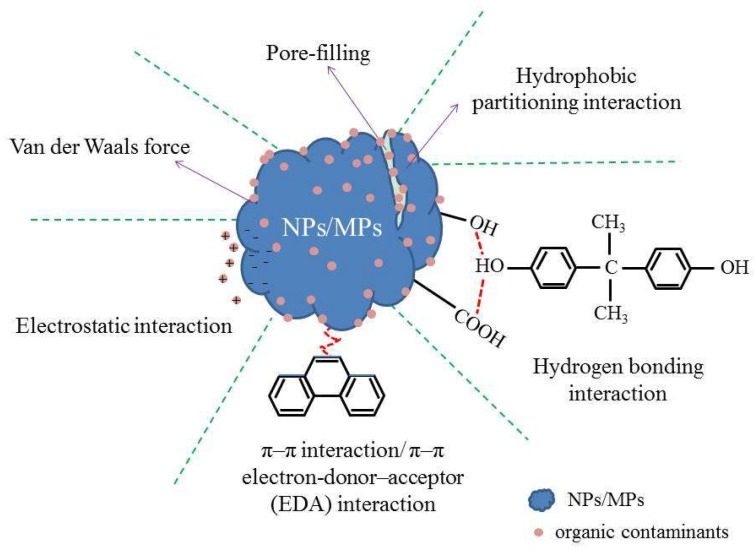
The sorption mechanisms of organic contaminants to NPs/MPs.

The sorption behaviors and mechanisms of organic contaminants to NPs/MPs in aquatic environments are listed in Table 1 and Figure 1.

### 2.1. Hydrophobic Partitioning Interaction

The hydrophobic partitioning interaction refers to the partition of organic compounds between the aqueous phase and NPs/MPs. The partitioning process is mainly characterized by the linear sorption isotherm [36]. Guo et al. [37] reported that sorption isotherms of phenanthrene, lindane, naphthalene, and 1-naphthol by PE were highly linear, for which the *n* values were in the range of 0.944−1, suggesting that hydrophobic partitioning was the dominant sorption process. Razanajatovo et al. [25] found that the main sorption mechanism of three drugs (sulfamethoxazole (SMX), propranolol (PRP), sertraline (SER)) on PE was hydrophobic partitioning interaction, and it was also found that the compounds with high hydrophobicity are more easily adsorbed on MPs. Wang et al. [31] reported that perfluorooctanesulfonate (PFOS) and perfluorooctanesulfonamide (FOSA) were adsorbed on PE, PS, and PVC MPs by hydrophobic partitioning interaction. Xu et al. [24] reported that the sorption of SMX by PE MPs could be well fitted by a linear model (R^2^ = 0.99), indicating that the sorption process was dominated by the hydrophobic partitioning interaction.

### 2.2. Surface Sorption

#### 2.2.1. Hydrogen Bonding Interaction

Hydrogen bonds are specific weak electrostatic interactions involving hydrogen ions H^+^, and can affect the sorption of polymers when proton donor and proton acceptor groups are involved [38]. The functional groups of NPs/MPs and the organic contaminants may affect the sorption by hydrogen bonding interaction. Zhang et al. [23] reported that the sorption of oxytetracycline (OTC) onto the MPs was enhanced by a hydrogen bonding mechanism because the surface of the weathered PS foam contains more carboxyl and ester carbonyl groups. Li et al. [20] found that the amide group (proton donor group) of polyamide (PA) and the carbonyl group (proton donor group) of amoxicillin (AMX), tetracycline (TC), and ciprofloxacin (CIP) could form hydrogen bonds, and this could enhance the sorption affinity. Liu et al. [39] reported that compared with low-density PE, liner low-density PE, high-density PE, PP, PS, polycarbonate, PVC, and polymethyl methacrylate, PA MPs showed the lowest hydrophobicity, whereas it showed the highest sorption capacity to hydrophobic 17β-Estradiol (E2), which was likely because PA could act as a hydrogen bond acceptor. Endo et al. [40] also found that PA had stronger sorption affinity for a variety of hydrogen bond donor compounds (hormones, pharmaceuticals, biocides, and dichlorodiphenyltrichloroethane (DDT)) than PE because of the formation of H-bonds between PA and the organic compounds. Liu et al. [41] reported that hydrogen bonding interaction was a possible mechanism for the sorption of CIP by aged PS and PVC MPs.

#### 2.2.2. π–π Interaction/π–π Electron-Donor–Acceptor (EDA) Interaction

For NPs/MPs with benzene rings in their structure, π–π interaction is also a key driving force that acts between aromatic molecules [33,38]. Hüffer et al. [42] studied the sorption behavior of seven aliphatic and aromatic organic compounds (n-hexane, cyclohexane, benzene, toluene, chlorobenzene, ethylbenzoate, naphthalene) to four kinds of MPs (PA, PS, PVC, PE) in aqueous solution, and reported that the sorption capacity of PS was the highest, which is likely because of the π–π interaction between the aromatic phenyl group of PS and the aromatic organic compound. Liu et al. [41] showed that the sorption capacity of PS for CIP could reach the highest earlier than PVC, probably because the π–π bond enhanced the interaction between MPs and CIP. The sorption capacity of PS for TC was greater than that of PE and PP. This may due to the presence of a benzene ring in both the TC and PS polymers, which enhanced sorption via polarity and π–π interaction [22]. Liu et al. [39] reported that compared with PVC, PS had a stronger sorption affinity to E2, probably due to the π–π interaction of the benzene ring between PS and E2. Velzeboer et al. [18] also found that PS NPs showed stronger sorption of PCBs than PE MPs, partially because of the benzene ring structure of PS.

The π–π EDA interaction is a special and noncovalent attraction between the electron donor and the electron acceptor. For example, Wang et al. [17] reported that π–π EDA interaction is also an important sorption mechanism between PS and nitrobenzene, because PS can act as a π-electron donor and nitrobenzene can act as stronger π-electron acceptor due to the strong electron-withdrawing nitro group and electron-depleted benzene ring.

#### 2.2.3. Electrostatic Interaction

Electrostatic interaction occurs when both MPs/NPs and organic contaminants have electric charges, and electrostatic sorption is generated when they have opposite electric charges, while electrostatic repulsion is generated when they have the same electric charges. Affected by pH and the point of zero charge (pH_pzc_), the adsorbent is positively charged when the pH is lower than the pH_pzc_ of the adsorbent. Otherwise, it is negatively charged. Li et al. [20] studied the sorption behavior of five antibiotics (sulfadiazine, AMX, TC, CIP, and trimethoprim (TMP)) to PE, PS, PP, PA, and PVC MPs under a freshwater system. In the freshwater system, sulfadiazine, AMX, TC, and TMP were in zwitterionic and anionic form, while CIP was in cationic form. The five MPs were all negatively charged because the actual pH was higher than the pH_pzc_ of the MPs. Therefore, the sorption capacity of the cationic CIP was enhanced on the negatively charged MPs by electrostatic attraction. Li et al. [43] reported that the sorption affinity of TCS decreased with the increase of pH when the pH value was between 6.0 and 11.0. This is likely because TCS is in an ionic form at this pH range, and the proportion of TCS increased with the increase of the pH value, thus electrostatic repulsion between TCS and negatively charged PS increased and reduced the sorption affinity.

#### 2.2.4. Van der Waals Force

Van der Waals force is the force between molecules. Guo et al. [37] reported that PEs are nonpolar aliphatic polymers without specific functional groups and could only interact with the four tested compounds (phenanthrene, naphthalene, lindane, and 1-naphthol) by van der Waals force. Hüffer et al. [42] also showed that seven aliphatic and aromatic adsorbates could be only adsorbed on aliphatic PE via the specific van der Waals force. Xu et al. [24] found that the sorption of SMX on PE was linear; however, both sulfamethoxazole and PE MPs were negatively charged under experiment conditions (pH 6.8), and meanwhile, PE MPs carried a net negative charge. Thus, the hydrophobic interaction and electrostatic interaction could not explain the sorption process of SMX sorption on PE, which may be attributed to van der Waals interaction.

### 2.3. Pore Filling

It was reported that there are many pores of different sizes in NP/MP materials [19,23,37], thus, the organic contaminants can enter the pores and may be trapped in the nano-scale pores of MPs. Guo et al. [37] found that organic carbon content-normalized sorption coefficients of cross-linking in 20% cross-linked PS for phenanthrene, lindane, naphthalene, and 1-naphthol was 1.5 times that of linear PS, probably due to the higher porosity of cross-linked PS. The pore volume of the cross-linked PS is as high as 0.514 cm^3^/g, while the linear PS is only 0.004 cm^3^/g. Therefore, the adsorbate molecules can be adsorbed to the nano-scale pores of cross-linked PS by pore filling. Zhang et al. [23] studied the sorption of OTC by beached and virgin PS foam, which also involved the pore-filling mechanism. The beached PS foam adsorbed more than the virgin PS foam, which may be attributed to the higher micropore area of the beached foam. The micropore area of the virgin PS foam was not detected, but the micropore area of the beached PS foam was 0.50 ± 0.02 m^2^/g. The average pore diameter of the virgin PS foam was five times more than the beached PS foam (5.1 ± 0.2 nm). Guo et al. [19] found that the pore diameter of MPs (PE, PP, PS, PVC) was smaller than the molecular size of TYL, which led to the inability of pollutants to enter the pore interior. Therefore, the intraparticle diffusion rate in the third stage is significantly lower than the surface diffusion rate in the second stage. Zhang et al. [44] reported that the sorption capacity of PS to OTC was significantly higher than that of PE, which was mainly because PS had more folds and pore structure than the PE particles.

## 3. Factors Influencing the Adsorption of Organic Contaminants by NPs/MPs

### 3.1. The Properties of NPs/MPs

#### 3.1.1. The Differences Between NPs And MPs/Particle Size

Usually, with the particle size decreasing, the specific surface area and the amount of sorption sites will increase, and thus enhance the sorption capacity. It was reported that the sorption capacity of NPs was higher, even reaching 1–2 orders of magnitude greater than that of MPs [18]. Zhang et al. [34] found that the sorption capacity of PP MPs for 3,6-dibromocarbazole and 1,3,6,8-tetrabromocarbazole increased with decreasing particle size. The reason was that the reduction in particle size increased the specific surface area of the MPs. Wang et al. [26] studied the BET specific surface area of PE, PS, and PVC, which also played an important role in the sorption of pyrene (Pyr) by MPs. The BET surface area of the three MPs followed the order of PE > PS > PVC, and the sorption capacity of Pyr was 164.5, 81.8, and 58.4 μg/g, respectively.

It is noted that some studies reported that the smaller size, especially the nano-scale size, enhanced the aggregation of the particles, and thus reduced the specific surface area [17]. Wang et al. [17] reported that the sorption of phenanthrene to PS NPs was significantly lower than that to MPs, and that the sorption of nitrobenzene was comparable between PS NPs and MPs. This could be attributed to the reduced effective sorption sites caused by NP aggregation.

#### 3.1.2. Polarity

A few studies have shown that the sorption behavior of organic contaminants on NPs/MPs are strongly correlated with the hydrophobicity of MPs [21,43,45]. For example, MPs are generally hydrophobic, so they adsorb hydrophobic organic contaminants more easily. However, the surface of weathered MPs which had been in natural environments showed increased polarity after introduction of oxygen-containing groups, and their affinity for hydrophobic organic contaminants was reduced [46,47]. Liu et al. [41] found that aging could change the properties of virgin MPs. The introduction of surface oxygen-containing functional groups increased the polarity of the MPs, which may be the reason for the increased sorption capacity of aged MPs PS and PVC for hydrophilic CIP. Müller et al. [48] also found that aged PS had reduced sorption of fuel-related water contaminants, including benzene, toluene ethyl benzene, and xylene, due to oxidation of the surface layer and increased polarity.

#### 3.1.3. Crystallinity

Crystallinity is a description of the structure of a polymer and an important feature that affects the sorption behavior of NPs/MPs [49]. The degree of crystallinity is related to the arrangement of the principal carbon chain. If the polymeric chain is more ordered and fixed, the crystallinity is higher. However, the more disordered the polymeric chain, the larger the proportion of amorphous areas [38]. In the crystalline region, high energy is necessary for absorbing chemicals, while in amorphous regions, the atoms can move more freely, favoring chemical absorption. With the increase of crystallinity, the ability and rate of sorption of contaminants by polymers decreases [50,51]. Guo et al. [37] found that the organic carbon content-normalized sorption coefficients of PEs on phenanthrene, lindane, and naphthalene increased with decreasing crystallinity.

#### 3.1.4. Glass Transition Temperature

Glass transition temperature affects MPs and contaminant sorption processes [52]. The polymer includes the crystalline region and the amorphous region. Sorption of hydrophobic organic contaminants usually occurs in the amorphous regions [53]. The MPs can be classified into a glassy state or a rubbery state according to the glass transition temperature. Some studies have shown that rubbery plastics (PP, PE) have a higher affinity for contaminants than glassy plastics (polyethylene terephthalate, PVC) [37,54,55]. PP and PE are soft MPs with strong sorption capacity for organic contaminants. This is because soft PP and PE have relatively low glass transition temperature and they in a rubbery state at normal temperature. The rubber state has high mobility, so they have high accessibility to hydrophobic organic compounds [56,57,58]. Glassy polymers have strong sorption sites due to the presence of internal pores (nanocavities), resulting in a slower release rate of hydrophobic organic compounds [53].

#### 3.1.5. Functional Groups of NPs/MPs

The functional groups of NPs/MPs can affect their sorption behavior. For example, highly aromatic PS shows stronger sorption affinity to PCBs due to hydrophobicity and π–π interactions compared to PE [34]. The sorption capacity of aromatic PS for polycyclic aromatic hydrocarbons is higher than that of other nonaromatic polymers (PE, PP, and PVC) [59]. Wang et al. [31] reported that the sorption affinity of FOSA was the highest on PE, followed by PVC and PS, which could be attributed to their different substituted atoms or groups. Li et al. [20] reported that the amide functional groups present in PA had higher sorption capacity for four antibiotics (TMP, CIP, AMX, and TC) than PE and PVC in the freshwater system. Liu et al. [39] reported that PA had the highest sorption capacity for E2 in ten different kinds of MPs due to the presence of an amide functional group.

The oxygen-containing functional groups on NPs/MPs can also act as H-bond acceptors and interact with water molecules (H bond donors), and thus form water clusters on the surface of NPs/MPs [40,60,61]. The formation of three-dimensional water clusters can reduce the accessibility of organic contaminants to the sorption domain of NPs/MPs, and compete with organic contaminants for sorption sites, thereby reducing their sorption affinities [59]. Huffer et al. [62] reported that the sorption of organic compounds by aged PS was lower than that of virgin PS due to the formation of water clusters on the aged PS.

### 3.2. Properties of Organic Contaminants

The properties of organic contaminants also affect sorption. The hydrophobicity and hydrophilicity, the surface charge, and the functional groups of the pollutants can affect their sorption behavior.

Organic contaminants can be identified by its octanol–water partition coefficient for its hydrophobicity and hydrophilicity. It has been demonstrated that hydrophobic interaction can be a main mechanism for sorption of NPs/MPs [63]; thus, organic contaminants with high hydrophobicity can be more readily adsorbed on NPs/MPs [46]. Razanajatovo et al. [25] also found that compounds (SMX, PRP, SER) with high hydrophobicity were easily adsorbed on PE MPs. Wu et al. [21] reported that the linear sorption coefficients (Log *K*_d_) of CBZ, EE2, TCS, and 4MBC on PE MPs were 191.4, 311.5, 5140, 53,225 L/kg, respectively, increasing in the same order as their octanol–water partition coefficients. Hüffer et al. [42] also found that the Log *K*_d_ of MPs was significantly correlated with the hydrophobicity of the organic contaminants (*p* < 0.05). Wang et al. [31] showed that the sorption affinity of FOSA to PE was higher than that of PFOS. This is because FOSA is a highly symmetrical molecule with very low polarity, and PFOS is more polar than FOSA due to the presence of sulfonic acid groups. Therefore, the sorption of FOSA on PE was enhanced by the polar–polar interaction.

Organic contaminants, especially ionic compounds, can show different ionization states under different aquatic conditions. Thus, the sorption of organic contaminants on the NPs/MPs can be affected by electrostatic interaction. Wang et al. [31] reported that FOSA could be adsorbed on PS, while PFOS could not, because electrostatic repulsion occurred between PS and PFOS, which were both with negatively charged. Razanajatovo et al. [25] found that the positively charged SER and PRP adsorbed on the negatively charged PE was enhanced by electrostatic attraction at pH 6.85 ± 0.04, and the sorption of negatively charged SMX on PE was lower due to electrostatic repulsion.

The functional groups of the pollutants could also affect their sorption behavior. For example, the nitro group could attract the π electron on MPs and enhance the π–π EDA interaction between the chemicals and MPs with aromatic benzene rings [17]. Wang et al. [31] reported that the sorption amount of FOSA on PE was higher than that of PFOS, and this could be attributed to the sulfonamide functional group on FOSA. Elizalde-Velázquez et al. [64] also reported that the sorption of diclofenac sodium salt to MPs was enhanced due to the amino group in its structure.

### 3.3. Aquatic Conditions

#### 3.3.1. pH Value

The dissociation constant (*p*K_a_) and the pH of the solution determine the charged state of the organic contaminants and NPs/MPs [65], and this can affect the sorption affinity via electrostatic interaction. Liu et al. [41] reported that the sorption efficiency of virgin PS increased with the increasing of pH value when it was lower than pH 5. This is likely because, in this pH range, MPs are negatively charged and CIP is positively charged, the high concentration of H^+^ in solution could inhibit the sorption of CIP^+^, and the sorption capacity increased with the decrease of H^+^ concentration. When the pH was further increased, the sorption efficiency rapidly decreased because CIP^+^ becomes CIP^−^, and electrostatic repulsion between CIP and MPs inhibited sorption. Zhang et al. [23] studied the effect of pH on the sorption of OTC onto beached PS MPs, and found the effect of pH was more obvious than that on virgin MPs. The maximum sorption of the beached MPs occurred at approximately pH 5, which corresponded to the highest ratio of OTC zwitterion in the solution (97.7%), and the pH_pzc_ of the beached MPs was 4.96; thus, the electrostatic repulsion was the lowest between the beached MPs and OTC at this pH. At pH < 5.0 or >5.0, the electrostatic repulsion was higher due to the similarly charged MPs and OTC. Xu et al. [22] reported that the electrostatic repulsion determined the sorption process of TC and MPs in an acidic or alkaline water environment. Because the *p*K_a_ values of TC were 3.30, 7.68, and 9.69, at pH < 4.0, TC was a cation, and PS, PP, and PE MPs were also positively charged. At pH > 8.0, TC was mainly present in an anionic form, and PS, PP, and PE MPs were also negatively charged.

#### 3.3.2. Ionic Strength

The ions presented in the aqueous environment may compete with the organic contaminants for sorption sites on NPs/MPs, and thus affect the sorption of organic contaminants by NPs/MPs. Liu et al. [41] found that with increasing NaCl concentration, the sorption affinity of CIP by virgin PVC and aged PVC decreased. This is because the increase of Cl ions increased the cohesive energy, the cohesive density made the individual PVC chains attractive, and then the free volume of PVC decreased. The reduction in free volume made diffusion into the material more difficult. A related study proved that with the increase of chloride ion concentration, the sorption of PCBs by virgin PVC decreased [66]. Zhang et al. [23] reported that the sorption of OTC by both virgin and beached PS foams decreased with increasing ionic strength of NaCl, CaCl_2_, or Na_2_SO_4_. This may be because Ca^2+^ and Na^+^ strongly compete for cationic exchange sites (i.e., carboxyl groups) on the microplastic surfaces [23]. Llorca et al. [32] also found that the sorption affinity of PFASs on MPs in seawater is weaker than that in freshwater because of the presence of salts in seawater.

The effect of ionic strength on the sorption of contaminants by NPs/MPs could be different between different contaminants. Wu et al. [21] demonstrated that salinity from 0.05 to 3.5% did not affect the sorption of 4MBC, CBZ, and EE2, but the sorption capacity of TCS increased, which may be related to the salting out effect. Bakir et al. [29] found that salinity had no significant effect on the sorption of phenanthrene on PE and PVC, but decreased the sorption of DDT with the increase of salinity. Studies on the sorption properties of MPs for PFOS and FOSA showed that the increase of ionic strength (CaCl_2_ and NaCl) had no effect on the sorption properties of FOSA, but increased PFOS in PE and PS [31]. Zhang et al. [67] found that the increase of NaCl concentration had little effect on the sorption of three musks to PP.

#### 3.3.3. DOM

**Figure 2:**
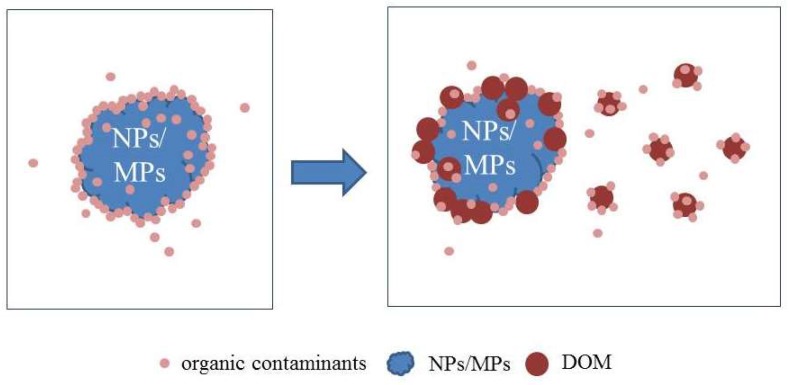
The sorption behavior of organic contaminants to NPs/MPs in the presence of dissolved organic matter (DOM).

DOM may affect the sorption of organic compounds by NPs/MPs through complex interactions (Figure 2). It was reported that the surface properties of NPs/MPs could be changed by the presence of DOM, and the dispersion stability of NPs was more affected by DOM than MPs [68]. Xu et al. [22] studied that fulvic acid had a significant effect on the sorption of TC by three MPs. When fulvic acid was added to 20 mg C/L, the adsorbed concentration of TC on the MPs was less than 10 μg/g, and the decreased percentage was more than 90%. This is likely because the affinity of TC on DOM was higher than that on MPs. Seidensticker et al. [69] reported that DOM could enhance the ability of hydrophobic organic contaminants desorbed from MPs. Zhang et al. [23] reported that with the increase of dissolved organic matter concentration, humic acid had a significant promotion effect on the sorption of OTC by beached MPs, possibly because humic acid acted as a bridge between the surface of the beached PS foam and the TC molecule.

#### 3.3.4. Coexisting Organic Contaminants

There are many pollutants in the aqueous environment, and the coexistence of multiple pollutants may result in competition for the sorption sites on NPs/MPs. Bakir et al. [70] studied the competitive sorption of persistent organic compounds (phenanthrene and DDT) on PE and PVC in the marine environment. In the marine environment, DDT sorption on the plastic surface was not affected by the presence of phenanthrene. Since the concentration of phenanthrene in the marine environment was higher than DDT, the phenanthrene content on the plastic surface was higher than DDT. When the initial addition of DDT concentration increased, DDT interfered with the sorption of phenanthrene, showing an antagonistic effect. Wang et al. [27] also found that the presence of Pyr had a negative impact on sorption of phenanthrene to PE, PS, PVC, and natural sediments, indicating a competitive relationship between contaminants.

## 4. Effect of NPs/MPs on Organic Contaminant Transport

Because of the strong sorption affinity to organic contaminants, NPs/MPs can act as carriers to migrate together with pollutants in the environment. Liu et al. [71] reported that PS NPs can significantly enhance the transport of the nonpolar contaminants Pyr and polar 2,2′,4,4′-tetrabromodiphenyl ether at low concentrations (5–20 mg/L); however, it had little effect on three polar compounds, bisphenol A, bisphenol F, and 4-nonylphenol. This is likely because desorption hysteresis on NPs occurs only in nonpolar/weakly polar contaminants. Nonpolar compounds are physically trapped in the internal matrix of PS glassy polymer structures, while polar compounds are more prone to surface sorption. Alimi et al. [72] demonstrated that the PU used in the repair paradigm improved the removal of phenanthrene in the soil by promoting the migration of contaminants in porous media and increasing the bioavailability of the microbial population. Substances with higher affinity organic matter may adhere more strongly to the suspended plastic particles and be transported by ocean currents to remote areas. Zarfl et al. [73] estimated that the quantity of plastic additives shipped to the Arctic Ocean is 250 g to 130 kg per year based on the estimated plastic flux to the Arctic Ocean ranging from 62,000 to 105,000 tons per year. Since the amount of MPs may exceed the actual amount of plastic collected, this may increase the amount of actual contaminants that plastics transport to the North Pole.

## 5. Outlook

Although there have been many studies on the mechanisms of organic contaminant sorption to NPs/MPs in recent years, the mechanisms controlling the sorption behavior between the organic contaminants and NPs/MPs are not fully understood yet. For example, current studies are mostly carried out indoors, in which the influencing factors are relatively simple compared the real environments. The complicated aquatic conditions in real environments could have combined effects which may differ from single influencing factors, and studies addressing this are still lacking. In addition, the properties of aged NPs/MPs in the real environment could be also different from those aged under simulated conditions; therefore, NPs/MPs from real environments could be selected for comparison. Meanwhile, a mathematic model should be established to predict the sorption of different organic contaminants to different NPs/MPs under various environmental conditions. This could be helpful for calculating the sorption affinity when the properties of organic contaminants and NPs/MPs are known before the experiments are performed. It is noted that NPs/MPs are easily ingested by organisms, and then enter the food chain through predation, and eventually enter the human body and threaten human health. Therefore, the desorption behavior of organic contaminants from NPs/MPs in organisms should be a focus. Overall, the study of mechanisms of organic contaminant sorption to NPs/MPs is of great importance for predicting the transport and fate of organic contaminants and NPs/MPs in the environment.

## Figures and Tables

**Table 1:** Sorption parameters of different organic contaminants on NPs/MPs.

**Compound**	**Sorbate**	**Sorbent**	**Sorption Capacity (mg/g)**	*K*_d_ (L/Kg) *	**Experimental Conditions**	**Mechanisms**	**Ref**
Antibiotics	Ciprofloxacin (CIP)	PA	2.20 ± 0.65	96.5 ± 7.8	25 °C, 180 rpm, 4 d	Pore filling, electrostatic interaction, hydrogen bonding	[20]
	Trimethoprim (TMP)	PA	0.468 ± 0.13	17.1 ± 1.2			
	Amoxicillin (AMX)	PA	22.7 ± 23	756 ± 48			
	Tetracycline (TC)	PA	3.84 ± 0.84	356 ± 38			
		PE	0.109	-	25 °C, 200 rpm, 24 h	Van der Waals force, electrostatic interaction, hydrophobic interaction, π–π interaction	[22]
		PP	0.113	-			
		PS	0.167	-			
	Oxytetracycline (OTC)	virginPS	1.52 ± 0.12	41.7 ± 5.0	25 ± 1 °C, 150 rpm, 54 h	Pore filling, electrostatic interaction, hydrogen bonding	[23]
		agedPS	27.5 ± 5.1	428 ± 15			
	Tylosin (TYL)	PE	1.67	62.8	25 °C, 150 rpm, 36 h	Electrostatic interaction, hydrophobic interaction, surface complexation	[19]
		PP	3.33	94.1			
		PS	3.33	134			
		PVC	3.33	155			
	Sulfamethoxazole (SMX)	PE	-	591 ± 24	25 °C, 200 rpm, 24 h	Van der Waals force, Partition interaction	[24]
	Carbamazepine (CBZ)	PE	-	191 ± 6.4	pH = 5, 5 d	Hydrophobic interaction	[21]
	Triclosan (TCS)	PE	-	5140 ± 290			
Other	4-Methylbenzylidene camphor (4MBC)	PE	-	53,200 ± 3700	5 d, pH = 5	Hydrophobic interaction	[21]
	Propranolol (PRP)	PE	-	2300 ± 2800	24 °C, 150 rpm, pH = 6.85, 96 h	Hydrophobic interaction, electrostatic interaction	[25]
	Sertraline (SER)	PE	-	3330 ± 800			
Polycyclic aromatic hydrocarbons (PAHs)	Pyrene (Pyr)	PE	0.165	-	25 °C, 200 rpm, 48 h	Hydrophobic interaction	[26]
		PS	0.0818	-			
		PVC	0.0584	-			
	Phenanthrene (Phe)	PE	0.714	3300	20 °C, 200 rpm, 48 h	Hydrophobic interaction	[27]
		PS	0.400	1860			
		PVC	0.303	1030			
		PE	-	38,100 ± 5600	18 °C, 200 rpm, 24 h	Hydrophobic interaction	[28]
		PP	-	2190 ± 170			
		PVC 200-500	-	1650 ± 200			
		PVC130	-	1690 ± 310			
		PVC	-	2000	18 °C, 220 rpm, 24 h	Pore filling, hydrophobic interaction	[29]
		PE	-	49800			
Polychlorinated biphenyls (PCBs)	3,3′,4,4′-tetrachlorobiphenyl (PCB77)	PP	0.350	1180	room temperature, 24 h	Hydrophobic interaction	[30]
Perfluoroalkyl substances (PFASs)	Perfluorooctanesulfonamide (FOSA)	PE	-	298	25 °C, 150 rpm, 7 d, pH = 7.0 ± 0.2	Hydrophobic interaction, Van der Waals force, electrostatic interaction	[31,32]
		PS	-	84.9			
		PVC	-	116			
		PS	-	-/619	20 °C, 120 rpm, 7 d, freshwater/seawater		
		PS-COOH	-	630/460			
	Perfluorooctanesulfonate (PFOS)	PE	-	32.8	25 °C, 150 rpm, 7 d, pH = 7.0 ± 0.2		
		PVC	-	101			
		PS	-	501/-	20 °C, 120 rpm, 7 d, freshwater/seawater		
		PS-COOH	-	101/-			
	perfluorobutanoic acid (PFBA)	PS	-	4.40/136			
		PS-COOH	-	9.33/197			
	perfluoropentanoic acid (PFPeA)	PS	-	12.0/270			
		PS-COOH	-	26.0/367			
		HDPE	-	-/76.9			
	perfluorobutanesulfonate (PFBS)	PS	-	27.7/140			
		PS-COOH	-	12.5/128			
	perfluorohexanoic acid (PFHxA)	PS	-	-/57.6			
		PS-COOH	-	-/64.0			
		HDPE	-	-/53.3			
	perfluoroheptanoic acid (PFHpA)	PS	-	-/53.9			
		HDPE	-	-/54.3			
	perfluorohexasulfonate (PFHxS)	PS	-	27.1/-			
		PS-COOH	-	2.21/-			
	Perfluorooctanoic acid (PFOA)	PS	-	8.37/-			
		HDPE	-	-/64.1			
	perfluorononanoic acid (PFNA)	HDPE	-	-/41.7			
	perfluorodecanoic acid (PFDA)	PS	-	455/466			
		PS-COOH	-	100/371			
	perfluorodecanesulfonate (PFDS)	PS-COOH	-	673/801			
	perfluoroundecanoic acid (PFUnA)	PS	-	-/565			
		PS-COOH	-	578/1430			
	perfluorododecanoic acid (PFDoA)	PS	-	-/525			
		PS-COOH	-	1210/2290			
	perfluorotridecanoic acid (PFTrA)	PS	-	1050/1450			
		PS-COOH	-	2070/4240			
		HDPE	-	596/-			
	Perfluorotetradecanoic acid (PFTeA)	PS	-	-/1220			
		PS-COOH	-	1770/-			
		HDPE	-	642/-			
	perfluorohexadecanoic acid (PFHxDA)	PS	-	396/-			
		PS-COOH	-	-/1930			
		HDPE	-	341/-			
	perfluorooctadecanoic acid (PFODA)	PS	-	515/1660			
		PS-COOH	-	833/803			
Pesticide	^14^C-DDT	PE	-	96,900 ± 21,000	18 °C, 48 h, seawater	Pore filling, partition interaction	[33]
Polyhalogenated carbazole (PHCs)	3,6-Dibromocarbazole (3,6-BCZ)	PP	0.402	-	220 rpm, 8 h	Surface sorption	[34]
	1,3,6,8-Tetrabromocarbazole (1,3,6,8-BCZ)	PP	0.415	-			
Endocrine disrupting chemicals (EDCs)	17α-Ethinyl estradiol (EE2)	PE	-	312 ± 21	5 d, pH = 5	Hydrophobic interaction	[21]
	Bisphenol A	PVC	0.190 ± 0.02	-	298 K, 200 rpm, 1100min, pH = 7	Hydrophobic interaction, electrostatic interaction, noncovalent bonds (hydrogen and halogen bonds)	[35]
	4,4′-Sulfonyldiphenol	PVC	0.150 ± 0.01	-			
	4,4′ Dihydroxydiphenylmethane	PVC	0.160 ± 0.01	-			
	2,2 bis(4 hydroxyphenyl) butane	PVC	0.220 ± 0.01	-			
	4,4′-(hexafluoroisopropylidene) diphenol	PVC	0.240 ± 0.02	-			

* *K*_d_ (the sorption coefficient) is defined as *K*_d_ = *q*/*C*_W_, where *q* and *C*_W_ are the equilibrium concentrations of an adsorbate on NPs/MPs and in the solution, respectively.
